# Tetrahydrobiopterin Administration Augments Exercise-Induced Hyperemia and Endothelial Function in Patients With Systemic Sclerosis

**DOI:** 10.3389/fmed.2021.791689

**Published:** 2022-01-10

**Authors:** Daniel R. Machin, Heather L. Clifton, D. Walter Wray, Tracy M. Frech, Anthony J. Donato

**Affiliations:** ^1^Department of Internal Medicine, University of Utah, Salt Lake City, UT, United States; ^2^Geriatric Research Education and Clinical Center, VA Salt Lake City, Salt Lake City, UT, United States; ^3^Department of Nutrition and Integrative Physiology, Florida State University, Tallahassee, FL, United States; ^4^Department of Nutrition and Integrative Physiology, University of Utah, Salt Lake City, UT, United States; ^5^Department of Internal Medicine, Vanderbilt University Medical Center, Nashville, TN, United States; ^6^Department of Biochemistry, University of Utah, Salt Lake City, UT, United States

**Keywords:** exercise, systemic sclerosis, arterial function, physiology, blood flow, vasodilation, handgrip

## Abstract

Systemic sclerosis (SSc) is a rare, auto-immune disease with variably progressive fibrosis of the skin and internal organs, as well as vascular dysfunction. Recently, we demonstrated a decrement in exercising skeletal muscle blood flow and endothelium-dependent vasodilation in SSc, but the mechanisms responsible for these impairments have not been investigated. Thus, we sought to determine if acute administration of tetrahydrobiopterin (BH_4_), an essential cofactor for endothelial nitric oxide synthase (eNOS), would improve hyperemia and brachial artery vasodilation during progressive handgrip exercise in SSc. Thirteen patients with SSc (63 ± 11 years) participated in this placebo-controlled, randomized, double-blind, crossover study. Tetrahydrobiopterin (10 mg/kg) administration resulted in a ~4-fold increase in circulating BH_4_ concentrations (*P* < 0.05). Cardiovascular variables at rest were unaffected by BH_4_ (*P* > 0.05). During handgrip exercise, BH_4_ administration increased brachial artery blood flow (placebo: 200 ± 87; BH_4_: 261 ± 115 ml/min; *P* < 0.05) and vascular conductance (placebo: 2.0 ± 0.8; BH_4_: 2.5 ± 1.0 ml/min/mmHg; *P* < 0.05), indicating augmented resistance artery vasodilation. Tetrahydrobiopterin administration also increased brachial artery vasodilation in response to exercise (placebo: 12 ± 6; BH_4_: 17 ± 7%; *P* < 0.05), resulting in a significant upward shift in the slope relationship between Δ brachial artery vasodilation and Δ shear rate (placebo: 0.030 ± 0.007; BH_4_: 0.047 ± 0.007; *P* < 0.05) that indicates augmented sensitivity of the brachial artery to vasodilate to the sustained elevations in shear rate during handgrip exercise. These results demonstrate the efficacy of acute BH_4_ administration to improve both resistance and conduit vessel endothelial function in SSc, suggesting that eNOS recoupling may be an effective strategy for improving vasodilatory capacity in this patient group.

## Introduction

Systemic sclerosis (SSc, scleroderma) is a rare auto-immune disease that is characterized by variably progressive fibrosis of the skin and internal organs, as well as an attenuated exercise capacity (1). Despite variability in the extent of organ involvement among SSc patients, the presence of enhanced peripheral vascular resistance is nearly universal (2). Indeed, a meta-analysis has reported that peripheral arterial vasodilatory capacity, as determined by flow-mediated dilation, is diminished in patients with SSc (3). Additionally, we have shown that the reactive hyperemic response during the flow-mediated dilation testing is also blunted in this population (4). Although the attenuated exercise capacity in SSc patients (1) is often linked to cardiopulmonary abnormalities (5, 6), exercise capacity remains impaired in SSc patients without central hemodynamic impairments (7), suggesting that impairments in peripheral vascular control play an important role in exercise intolerance in this patient group.

We have recently demonstrated marked impairments in the peripheral vascular response to exercise in SSc patients (8). Compared to healthy controls, we observed a ~35% reduction in forearm blood flow and vascular conductance during progressive handgrip exercise in SSc patients, demonstrating a clear disease-related impairment in “exercise hyperemia” in this patient group. This decrement in resistance vessel responsiveness was accompanied by blunted dilation of the brachial artery in response to the sustained elevation in shear rate present during exercise, suggesting that conduit vessel endothelium-dependent vasodilation is also diminished in SSc patients. Considering the widespread prevalence of elevated peripheral vascular resistance and diminished vasodilatory capacity in SSc, it is likely that dysfunctional peripheral arteries contribute to the attenuated exercise capacity in this population.

In addition to peripheral vascular dysfunction, blood concentrations of oxidative stress and damage markers are elevated in patients with SSc (9, 10). The vascular endothelium is particularly vulnerable to oxidative damage (11), as increases in oxidative stress result in endothelial dysfunction (12) that can impair the peripheral vascular response to exercise (13). Indeed, we have shown that elevated blood oxidative stress markers accompany brachial arterial endothelial dysfunction during handgrip exercise in SSc patients (8). Therefore, if brachial artery endothelial dysfunction during exercise is due to elevated systemic oxidative stress then it is likely that the downstream resistance artery vasodilatory dysfunction may also be due to oxidative stress-induced endothelial dysfunction.

Low endothelial tetrahydrobiopterin (BH_4_) bioavailability is one potential source of oxidative stress in SSc patients. Tetrahydrobiopterin is an essential cofactor for endothelial nitric oxide synthase (eNOS) (14) that is critical for maintaining nitric oxide (NO) bioavailability in the vascular endothelium (15). An insufficient endothelial concentration of BH_4_ results in “uncoupled” eNOS that no longer produces NO, but produces superoxide instead (16). Increased superoxide can lead to peroxynitrite formation, which, in turn, oxidizes BH_4_ to its inactive form, leading to further eNOS uncoupling, greater superoxide formation, and reduced NO bioavailability (17). We have recently reported that acute BH_4_ administration augments resting brachial artery flow-mediated dilation and reactive hyperemia in SSc patients (18). Currently, it is unknown if exogenous BH_4_ administration can improve the peripheral vascular response to progressive handgrip exercise in SSc patients. Therefore, we sought to examine the peripheral vascular response to progressive handgrip exercise after acute oral BH_4_ administration in patients with SSc. Progressive handgrip exercise was employed in this study, as it incorporates a small muscle mass, thus, requiring a fraction of maximal cardiac output, limiting the impact of central hemodynamic factors (19). We hypothesized that, compared to placebo, acute BH_4_ administration would augment exercise-induced hyperemia, our primary endpoint, as well as the brachial artery vasodilation to increases in shear rate that occur during handgrip exercise.

## Materials and Methods

### Ethical Approval

Written informed consent was obtained prior to participation after an explanation of the nature, benefits, and risks of the study. All procedures were approved by the institutional review board of the University of Utah and Salt Lake City Veterans Affairs Medical Center (IRB# 38705), which serves as the ethics committee.

### Study Participants

Thirteen patients with SSc were recruited from the University of Utah SSc Clinic to participate in this study. Patients were previously diagnosed with SSc, by 2013 classification criteria (20). Body mass index was calculated from height and body mass. The clinical features of patients with SSc that were recorded for SSc disease duration, cardiovascular-acting medications, SSc-related vasculopathy medical history (i.e., pulmonary arterial hypertension, scleroderma renal crisis, and/or digital ulcer), antinuclear antibody, and SSc-specific antibody status. None of the participants had diabetes mellitus, overt cardiovascular disease, or met criteria for another inflammatory overlap rheumatic disease.

### Experimental Design

A placebo-controlled, randomized, double-blind, crossover experimental design was employed with a washout period of at least 5 days before crossing over into the alternate study drug condition. Treatment randomization was performed by DWW using coin flip and kept confidential from the remainder of the research team. On the experimental days, patients reported to the laboratory after having consumed a standardized breakfast and oral BH_4_ (10 mg/kg) or placebo in tablet form 5 h prior to their arrival. Main known side effects of BH_4_, such as headache and rhinorrhea, and participants were monitored for these side effects. Others have shown that this dose of oral BH_4_ administration effectively increases circulating BH_4_ concentrations (21, 22). Participants were instructed to abstain from food (not including the standardized breakfast), alcohol, caffeine, and exercise for ≥12 h prior to arrival. Additionally, vasodilatory medications were discontinued 12 h prior to study visit. In premenopausal women, measurements were performed during the early follicular phase of the menstrual cycle. All measurements were made under quiet, comfortable, ambient (~22°C) laboratory conditions at the same time of day to eliminate any diurnal effects. The primary endpoint measure was brachial artery blood flow in response to progressive handgrip exercise measured via ultrasound Doppler. Secondary endpoint measures were forearm vascular conductance and brachial artery vasodilation in response to progressive handgrip exercise measured via ultrasound Doppler.

### Progressive Handgrip Exercise

After collection of a venous blood sample, participants were instrumented for assessment of heart rate and arterial blood pressure. Static intermittent handgrip exercise was then performed at 1 Hz, as described previously (19). Participants were encouraged to perform rapid contractions with the goal of limiting contraction time to <25% of the duty cycle. Participants exercised at 15, 30, and 45% of their maximum voluntary contraction. Each exercise stage was performed for 3 min with a 2-min break allotted between each workload.

### Measurements

Heart rate was monitored with a three-lead electrocardiogram, recorded in duplicate on the data acquisition device (Biopac, Goleta, CA) and ultrasound Doppler (Logic 7, GE Medical Systems, Milwaukee, WI). Mean arterial blood pressure (MAP) was measured in the non-exercising contralateral arm by auscultation of the brachial artery (Tango+, SunTech, Morrisville, NC). Simultaneous measurements of brachial artery blood velocity and vessel diameter were performed using a linear array transducer operating in duplex mode, with imaging frequency of 14 MHz and Doppler frequency of 5 MHz. The brachial artery was insonated approximately midway between the antecubital and axillary regions, medial to the biceps brachii muscle. All measurements were obtained with the probe appropriately positioned to maintain an insonation angle of ≤ 60°. The sample volume was maximized according to vessel size and was centered within the vessel based on real-time ultrasound visualization. Angle-corrected, time-average, and intensity-weighted mean blood velocity values were calculated using commercially available software (Logic 7). Brachial artery vasodilation was determined offline from end-diastolic, ECG R-wave-triggered images collected from the ultrasound Doppler using automated edge-detection software (Medical Imaging Applications, Coralville, IA). Ultrasound Doppler measurements were performed continuously, with the last 60 s of each exercise intensity used for the determination of limb blood flow.

Mean arterial blood pressure was calculated according to the equation: MAP (mmHg) = systolic blood pressure · 1/3 + diastolic blood pressure · 2/3. Shear rate was calculated according to the equation: shear rate (s^−1^) = blood velocity · 4/vessel diameter. Brachial artery blood flow was calculated as per the equation: blood flow (ml/min) = (blood velocity · π · [vessel diameter/2]^2^ · 60). Brachial artery vascular conductance was calculated according to the equation: blood flow/MAP.

### BH_4_ Quantification

Blood was collected in EDTA plasma tubes containing 1 mM dithiothreitol (DTE) in 0.9% saline to give a final concentration of 0.1 mM DTE. After blood collection, tubes were centrifuged at 3,000 RPM for 15 min at 4°C, and plasma was collected and stored at −80°C. To quantify BH_4_ concentrations, plasma was thawed and extracted using differential oxidation with iodine using the Fukushima-Nixon method (23), which enables measurement of total biopterin and BH_4_. Under acidic conditions, BH_4_ and 7,8-dihydrobiopterin (BH_2_) are oxidized to biopterin. Under alkaline conditions only BH_2_ is oxidized to biopterin, while BH_4_ undergoes side-chain cleavage to form pterin. Tetrahydrobiopterin levels was quantified by calculating the difference in biopterin content between the two oxidation reactions. Prior to iodine oxidation, the plasma was deproteinized by adding 250 μl of 1 M trichloroacetic acid to 1 ml plasma. This was incubated in the dark at 4°C for 15 min and centrifuged at 20,000 g for 15 min at 4°C. HPLC of biopterin was performed on an Acquity Arc system with a CORTECS®C18, 2.7 μm column (4.6 × 150 mm) with a Vanguard® pre-column at a column temperature of 40°C. All were from Waters Corporation. Samples were run isocratically with 15 mM potassium phosphate buffer, pH 6.4 at a flow rate of 0.8 ml/min. A Waters 2475 Multi λ Fluorescence Detector was used to detect biopterin with excitation set to 350 nm and an emission setting of 440 nm.

### Statistical Analyses

Statistical analyses were performed using GraphPad Prism (GraphPad Software, San Diego, CA). Paired *t*-tests were used to compare differences in participant characteristics, cardiovascular variables at rest. A two-way repeated-measures ANOVA was used to evaluate differences between placebo and BH_4_ during exercise, and a least significant difference paired *t*-test identified the means that were significantly different. Univariate linear regression analysis was performed to confirm associations between Δ brachial artery vasodilation and Δ shear rate. Multiple linear regression was performed to model the relationships of placebo and BH_4_ with Δ brachial artery vasodilation to Δ shear rate. Slopes were compared by applying analysis of covariance (ANCOVA) to the straight lines obtained by the regression methods (24). Statistical significance was set at *P* < 0.05 for all analyses. Data are presented as mean ± SD.

## Results

### Participant Characteristics

Participant characteristics, including SSc-related vasculopathy medical history, are presented in Table 1. Among these participants, SSc disease duration ranged from 1 to 36 years with a mean 7 ± 11 years. Nearly all the SSc patients (85%) were prescribed calcium channel blockers as well as other vasodilatory medications, all of which were discontinued 12 h prior to the study visits. The majority of the SSc patients had a history of digital ulcers (54%), while two of these patients with a positive digital ulcer history also had pulmonary arterial hypertension or scleroderma renal crisis. Antinuclear antibody testing was previously performed in all but one of the SSc patients. All the SSc patients that were tested were positive for antinuclear antibodies and the majority (58%) were positive for anti-centromeric antibodies.

**Table 1:** Participant characteristics.

**Women:men**	**9:4**
**Age, years**	**63 ± 11**
**Height, cm**	**170 ± 10**
**Weight, kg**	**70.3 ± 11.0**
**Body mass index, kg/m^2^**	**24.3 ± 2.5**
**Maximum voluntary contraction, kg**	**17.5 ± 6.7**
**SSc disease duration, years**	**7 ± 11**
**Cardiovascular-acting medications, *n* (%)**	
**Calcium channel blockers**	**11 (85)**
**Endotdelin receptor antagonists**	**0 (0)**
**Phosphodiesterase inhibitors**	**2 (15)**
**SSc-related medical history, *n* (%)**	
**Digital ulcer**	**7 (54)**
**Pulmonary arterial hypertension**	**2 (15)**
**Scleroderma renal crisis**	**1 (8)**
**Antibody presence^a^, *n* (%)**	
**Antinuclear antibody**	**12 (100)**
**Centromere**	**7 (58)**
**RNA polymerase III**	**1 (8)**
**SCL70**	**2 (17)**
**Fibrillin**	**2 (17)**
**RNP**	**1 (8)**

*SSc, systemic sclerosis. Values are presented as mean ± SD.*

a *Antibody presence was tested in 12 patients*.

### Circulating BH_4_ Concentrations

Tetrahydrobiopterin administration increased plasma BH_4_ concentrations ~4-fold compared with placebo (placebo: 10.9 ± 2.2; BH_4_: 39.5 ± 13.0 nmol/L; *P* < 0.05). Blood markers of oxidative stress, antioxidant capacity, and inflammation in this cohort have been published elsewhere (18), and were unchanged between placebo and BH_4_ conditions.

### Effects of BH_4_ at Rest

There was no effect of BH_4_ on handgrip maximum voluntary contraction (placebo: 17.9 ± 7.2; BH_4_: 17.4 ± 6.1 kg; *P* > 0.05). Similarly, cardiovascular variables were unaffected by acute BH_4_ administration at rest (Table 2). No patient reported any side effect or adverse events in response to acute BH_4_ administration.

**Table 2:** Cardiovascular variables at rest and during exercise.

	**Exercise Intensity**			
**Relative % of max**	**Rest**	**15**	**30**	**45**
**Placebo**				
Heart rate, bpm	68 ± 9	71 ± 10	73 ± 9	76 ± 10
Systolic blood pressure, mmHg	114 ± 11	125 ± 18	131 ± 18	140 ± 18
Diastolic blood pressure, mmHg	70 ± 7	78 ± 7	78 ± 7	83 ± 7
Lumen diameter, mm	3.23 ± 0.58	3.38 ± 0.59	3.45 ± 0.62	3.6 ± 0.62
Blood velocity, cm/s	4.2 ± 1.0	18.8 ± 6.4	27.3 ± 6.8	31.5 ± 7.4
Shear rate, s^−1^	55 ± 18	232 ± 95	329 ± 115	361 ± 109
**BH**_**4**_				
Heart rate, bpm	66 ± 8	70 ± 10	72 ± 9	78 ± 8
Systolic blood pressure, mmHg	112 ± 14	125 ± 14	131 ± 14	143 ± 18
Diastolic blood pressure, mmHg	70 ± 7	77 ± 7	79 ± 7	83 ± 7
Lumen diameter, mm	3.28 ± 0.56	3.53 ± 0.53^*^	3.65 ± 0.56^*^	3.81 ± 0.54^*^
Blood velocity, cm/sec	4.5 ± 1.4	22.5 ± 9.3^*^	31.2 ± 9.2^*^	37.3 ± 10.1^*^
Shear rate, s^−1^	57 ± 25	261 ± 112^*^	351 ± 115^*^	399 ± 115^*^

*Values are presented as mean ± SD.*

* *P < 0.05 vs. placebo*.

### Effects of BH_4_ During Exercise

**Figure 1:**
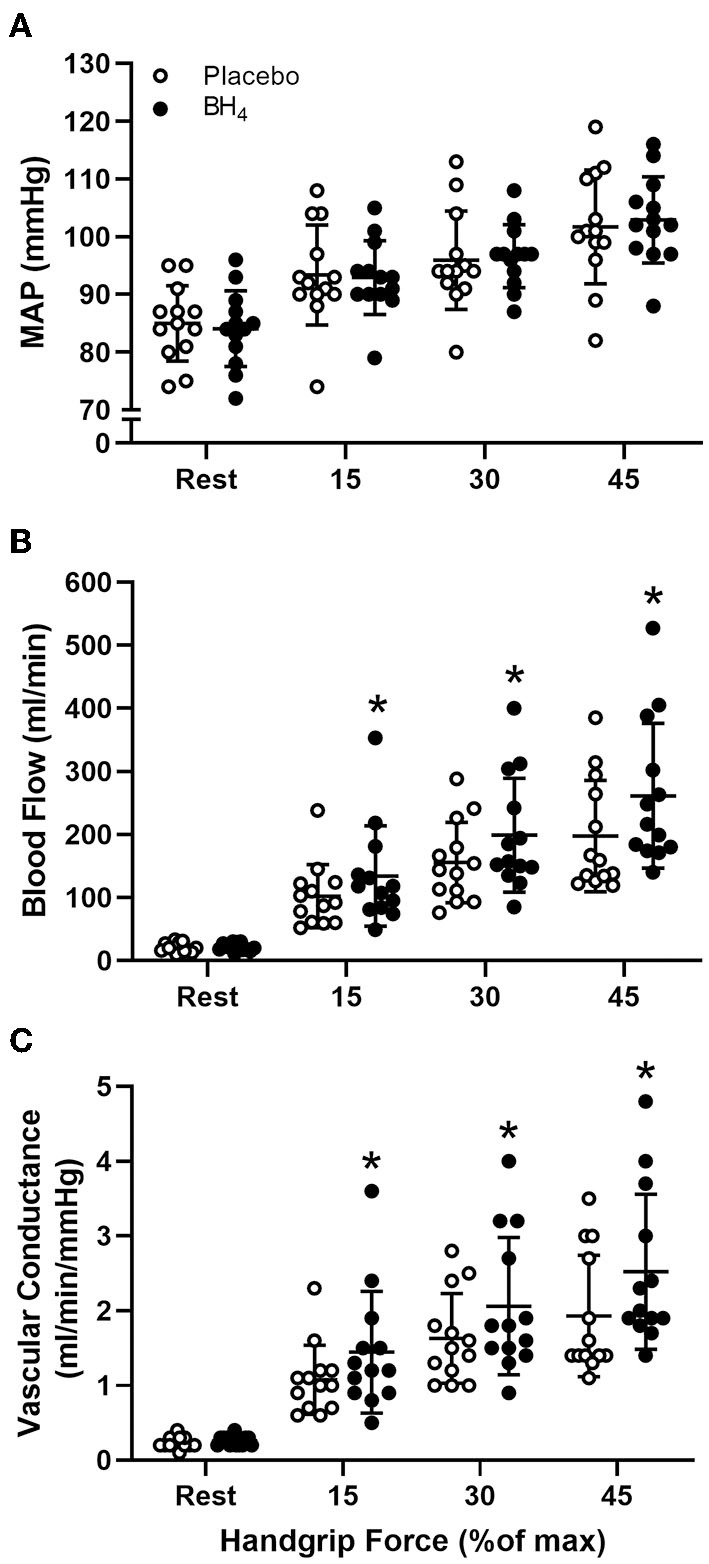
Mean arterial pressure [MAP **(A)**], brachial artery blood flow **(B)**, and brachial artery vascular conductance **(C)** at rest and during progressive handgrip exercise in patients with systemic sclerosis after placebo (white circles) and tetrahydrobiopterin (BH_4_; black circles). *P < 0.05, significantly different than placebo. All data are presented as mean ± SD.

**Figure 2:**
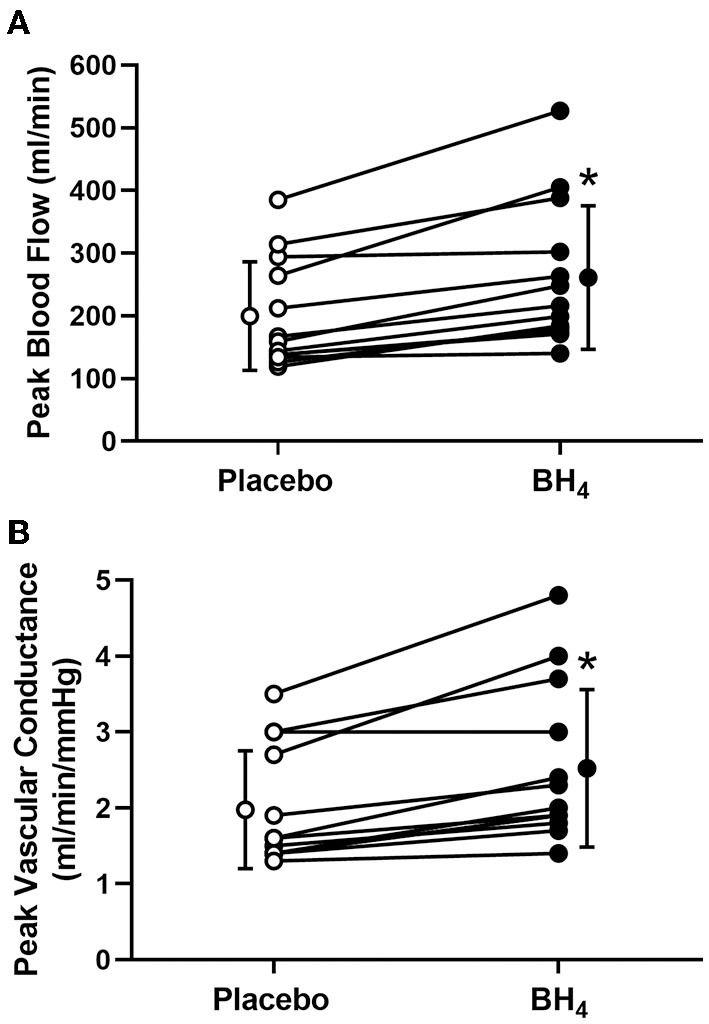
Individual peak brachial artery blood flow **(A)** and vascular conductance **(B)** in response to progressive handgrip exercise in patients with systemic sclerosis after placebo (white circles) and tetrahydrobiopterin (BH_4_; black circles). *P < 0.05, significantly different than placebo. Group data are presented as mean ± SD.

Heart rate and MAP increased with each workload in response to handgrip exercise but were not different between placebo and BH_4_ (*P* > 0.05; Table 2; Figure 1A). At each handgrip workload, exercise-induced brachial artery blood velocity was greater after acute BH_4_ compared to placebo (*P* < 0.05; Table 2). Exercise-induced brachial artery blood flow was ~28-32% greater after acute BH_4_ administration compared to placebo (*P* < 0.05; Figure 1B). Because MAP was not different between placebo and BH_4_, like blood flow, exercise-induced vascular conductance was ~28–34% greater at each handgrip workload after BH_4_ compared to placebo (*P* < 0.05; Figure 1C). Individual peak brachial artery blood flow and vascular conductance responses to handgrip exercise are presented in Figure 2. Eleven of the 13 participants had at least a 10% increase in peak brachial artery blood flow and vascular conductance after BH_4_ compared to placebo.

**Figure 3:**
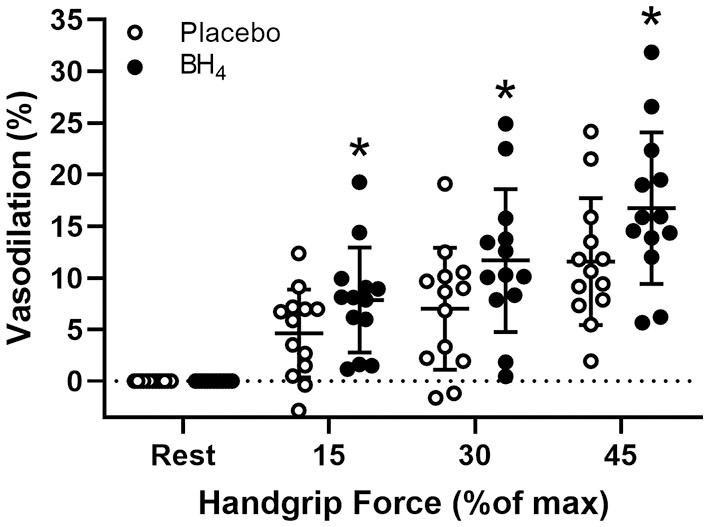
Brachial artery vasodilation during progressive handgrip exercise in patients with systemic sclerosis after placebo (white circles) and tetrahydrobiopterin (BH_4_; black circles). *P < 0.05, significantly different than placebo. All data are presented as mean ± SD.

**Figure 4:**
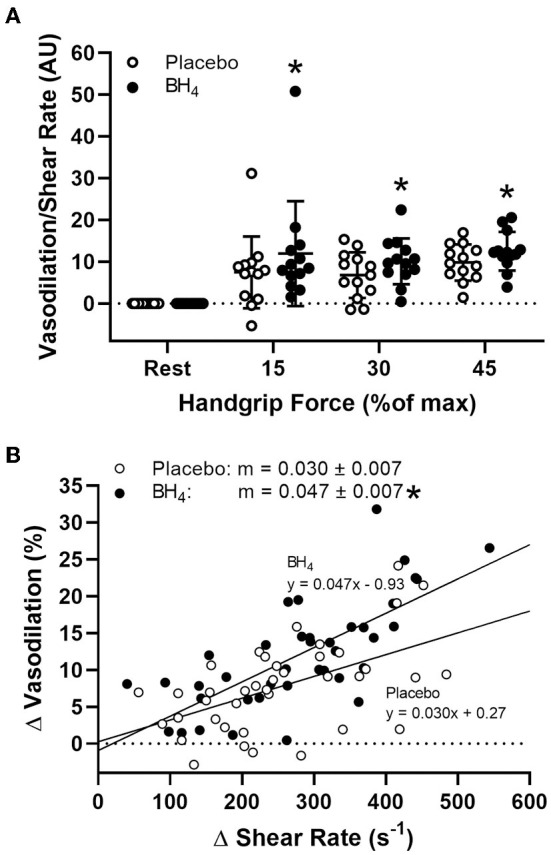
Brachial artery vasodilation normalized to increases in shear rate **(A)** during progressive handgrip exercise in patients with systemic sclerosis after placebo (white circles) and tetrahydrobiopterin (BH_4_; black circles). Brachial artery vasodilation to sustained increases in shear rate after placebo and BH_4_ **(B)**. After acute BH_4_ administration, patients with systemic sclerosis had a significantly higher slope (m) compared to placebo. *P < 0.05, significantly different than placebo. All data are presented as mean ± SD.

In response to handgrip exercise, brachial artery lumen diameter increased at each exercise workload (Table 2). Acute BH_4_ administration resulted in a ~45–70% greater vasodilatory response at each handgrip workload (*P* < 0.05; Figure 3). Exercise-induced shear rate was also significantly at each handgrip workload (*P* < 0.05; Table 2). Despite elevations in shear rate after BH_4_, Δ brachial artery vasodilation normalized to Δ shear rate was ~49–60% greater at each handgrip workload after BH_4_ administration compared to placebo (*P* < 0.05; Figure 4A), resulting in a significant upward shift in slope of the relationship between Δ brachial artery vasodilation and Δ shear rate (*P* < 0.05; Figure 4B).

## Discussion

The current study provides evidence that acute oral BH_4_ administration ameliorates the dysfunctional peripheral vascular response to exercise in SSc patients. The main findings are that, compared to placebo, BH_4_ administration increased circulating BH_4_ concentrations by ~4-fold, and had a positive effect on our primary endpoint, exercise-induced hyperemia, resulting in a greater brachial artery blood flow at all exercise workloads, indicating an improvement in resistance artery vasodilatory function. Acute BH_4_ administration also augmented vascular conductance, as well as the sensitivity of the brachial artery to vasodilate to the sustained elevations in shear rate that occur during handgrip exercise, indicating improved conduit artery endothelial function. Taken together, these results demonstrate the efficacy of acute BH_4_ administration to improve both resistance and conduit vessel endothelial function in SSc patients, suggesting that eNOS recoupling may be an effective strategy for improving vasodilatory capacity in this patient group.

### Role of BH_4_ on Resistance Vessel Function

In the current study, we observed that acute oral BH_4_ administration augments exercise-induced hyperemia in patients with SSc. Because BH_4_ had minimal effects on blood pressure at rest or during exercise, the magnitude of improvement in exercise-induced brachial artery vascular conductance was nearly identical to the improvement in exercise-induced hyperemia (Figure 1). The ability of resistance arteries to vasodilate during exercise is demonstrated by increases in blood flow and vascular conductance (25), both of which are partially mediated by NO (26, 27). Thus, one of the likely mechanisms for BH_4_-mediated improvements in exercise-induced blood flow and vascular conductance in this study are due to augmented resistance artery endothelial function. Although the role of NO in the regulation of exercising skeletal muscle blood flow continues to be debated, using an identical protocol to that employed in the present study, our group has previously identified a significant contribution of this pathway to the overall hyperemic response during handgrip exercise in young, healthy adults (19). Specific to SSc, we have recently shown that acute BH_4_ administration results in a slight, but significant, increase in brachial artery peak reactive hyperemia after 5 min of distal cuff occlusion in SSc patients (18). Like exercise hyperemia, the entirety of the reactive hyperemic response also cannot be attributed to NO-mediated resistance artery vasodilation, although NO does have a slight impact on the magnitude of reactive hyperemia (28–30). Still, as we did not employ any NO blockade in the current study, an improvement in resistance artery endothelial function via BH_4_-mediated improvement in NO signaling cannot definitively be shown in the current study.

### Role of BH_4_ on Conduit Vessel Function

During progressive handgrip exercise, stepwise increases in steady-state brachial artery blood flow result in sustained elevations in shear rate (31). It is now well-accepted that changes in shear rate are the stimulus for conduit arterial endothelium-dependent dilation (32). Therefore, brachial artery vasodilation during progressive handgrip exercise can be used as a measurement of endothelium-dependent dilation. Our group has utilized this ‘sustained stimulus flow-mediated dilation' in a variety of populations to detect endothelial dysfunction in both health and disease (8, 13, 33, 34), and importantly, have identified the NO-dependent nature of brachial artery vasodilation using this experimental model (19).

In SSc patients, we have reported that acute BH_4_ administration augments brachial artery flow-mediated dilation, which support the findings of the current study by demonstrating the beneficial effects of BH_4_ using a different model of conduit artery endothelium-dependent dilation (18). Therefore, it is likely that improved exercise-induced brachial artery vasodilation after acute BH_4_ administration in SSc patients observed in the present study (Figure 3) was the consequence of an NO-dependent improvement in endothelial function.

Given the known stimulus-response relationship between shear stress and endothelium-dependent vasodilation (35), it could be argued that augmented brachial artery vasodilation following BH_4_ administration was simply the consequence of a greater shear stimulus, as BH_4_ also increases exercise-induced blood flow. To explore this possibility, we normalized brachial artery vasodilation to changes in shear rate. When responses are viewed in this manner, a clear treatment effect of BH_4_ remains, as quantified by a steeper slope of vasodilation normalized to shear rate compared to placebo (Figure 4). This is particularly important given our previous findings that SSc patients demonstrate impaired brachial artery vasodilation to increases in shear rate during handgrip exercise (8) and in response to reactive hyperemia following ischemic cuff occlusion (4), and is suggestive that BH_4_ administration may act to partially restore conduit vessel endothelial function in this patient group. These findings both confirm and extend reported improvements in conduit (36–39), resistance (40–44), and coronary (45, 46) artery vasodilatory function with BH_4_ administration in a variety of populations, showing for the first time BH_4_-mediated improvements also occur during exercise.

### Mechanistic Insights

We have previously observed that elevated blood oxidative stress markers accompany the impaired vascular response to handgrip exercise in SSc patients (8). Although the etiology of disease-related increases in oxidative stress and damage in this population is not well-understood, deficits in endothelial BH_4_ may be a significant contributor, as uncoupled eNOS produces superoxide rather than NO (16). At supraphysiological concentrations, BH_4_ has been reported to have antioxidant properties, acting as a free radical scavenger *in vitro* (47, 48). However, it is unlikely that improvements in the vascular response to exercise with BH_4_ observed in the present study were due to an antioxidant effect, as BH_4_-mediated superoxide scavenging is not a major reaction *in vivo* (49). It is also unlikely that BH_4_ acted directly to reduce vascular free radical concentration. In fact, there is no data that suggests BH_4_ administration can acutely lower oxidative stress in any human population, and we have previously reported no changes in blood oxidative stress markers after BH_4_ administration in SSc patients (18). Thus, the most likely mechanism by which vascular function improved in the present study is a decline in superoxide concentration due to greater NO synthesis (50) that reflects eNOS recoupling. Although it appears acute BH_4_ administration does not lower oxidative stress to a magnitude of physiological significance, it should be noted that chronic BH_4_ supplementation does lower blood oxidative damage markers in hypercholesterolemic individuals (51). Thus, the long-term effects of BH_4_ are likely due to a chronic increase in BH_4_ bioavailability that permits greater eNOS coupling, thereby chronically lowering superoxide production and mitigating subsequent oxidative damage.

It is unlikely that BH_4_-mediated improvements were due to greater smooth muscle vasoreactivity, as others have reported endothelium-independent dilation to sublingual nitroglycerin to be unchanged with acute BH_4_ administration (36–40, 43, 44). Furthermore, we have previously observed no changes in brachial artery vasodilation or peak blood flow in response to sublingual nitroglycerin after acute BH_4_ administration in SSc patients (18). Taken together, these studies suggest that the beneficial effects of BH_4_ toward the vascular response to exercise come through improvements in endothelial function.

In contrast to handgrip exercise, there was no noticeable BH_4_-related effect on any cardiovascular variable at rest. There have also been no reported effects of acute BH_4_ administration on resting cardiovascular variables in other populations (36–39, 44). Thus, it is likely that the vascular improvements with BH_4_ are only exerted or noticeable when eNOS activation is elevated, such as in response to the increases in shear rate that arise with exercise or other physiological states that result in augmented blood flow.

## Conclusions

We have shown that acute BH_4_ administration improves both resistance and conduit artery vasodilatory function during exercise in patients with SSc. Tetrahydrobiopterin-mediated improvements were achieved despite no changes in blood oxidative stress markers, which we and others have shown to be elevated in SSc patients. Taken together, these results imply that improvements in the peripheral vascular response to exercise in SSc patients with BH_4_ were due to an improved endothelial function that was likely achieved though BH_4_-mediated eNOS recoupling. Moreover, because we did not observe any changes in cardiovascular hemodynamics or blood pressure at rest, these data provide the first initial evidence for the safe and effective use of BH_4_ in SSc. Thus, future studies are warranted to assess the chronic effects BH_4_ and its role as an add-on therapy to normally prescribed medications in this population (i.e., calcium channel blockers) in SSc patients.

## Data Availability Statement

The data that support the findings of this study are available from the corresponding author upon reasonable request.

## Ethics Statement

The studies involving human participants were reviewed and approved by Institutional Review Board of the University of Utah and Salt Lake City Veterans Affairs Medical Center (IRB# 38705). The patients/participants provided their written informed consent to participate in this study.

## Author Contributions

DRM, HLC, and TMF: performed experiments. DRM: prepared figures. DRM, HLC, DWW, and AJD: drafted manuscript. All authors analyzed data, conception, design of research, interpreted results of experiments, edited, revised manuscript, and approved the final version of manuscript.

## Funding

This work was supported by awards from the National Institutes of Health (R00 AT010017, R01 AG040297, P01 HL091830, R21 AG043952, K02 AG045339, and K23 AR067889) and the U.S. Department of Veterans Affairs (I01 RX001697, I01 CX001183, I01 RX001311, and I01 CX002152).

## Conflict of Interest

The authors declare that the research was conducted in the absence of any commercial or financial relationships that could be construed as a potential conflict of interest.

## Publisher's Note

All claims expressed in this article are solely those of the authors and do not necessarily represent those of their affiliated organizations, or those of the publisher, the editors and the reviewers. Any product that may be evaluated in this article, or claim that may be made by its manufacturer, is not guaranteed or endorsed by the publisher.
